# Circadian Clock Genes Are Correlated with Prognosis and Immune Cell Infiltration in Colon Adenocarcinoma

**DOI:** 10.1155/2022/1709918

**Published:** 2022-01-25

**Authors:** Aoxiao He, Zhihao Huang, Rongguiyi Zhang, Hongcheng Lu, Jiakun Wang, Jiaqing Cao, Qian Feng

**Affiliations:** ^1^Department of General Surgery, The Second Affiliated Hospital of Nanchang University, Nanchang 330000, China; ^2^Department of Emergency, The Second Affiliated Hospital of Nanchang University, Nanchang 330000, China

## Abstract

**Background:**

Colon adenocarcinoma (COAD) is a malignancy with a high incidence and is associated with poor quality of life. Dysfunction of circadian clock genes and disruption of normal rhythms are associated with the occurrence and progression of many cancer types. However, studies that systematically describe the prognostic value and immune-related functions of circadian clock genes in COAD are lacking.

**Methods:**

Genomic data obtained from The Cancer Genome Atlas (TCGA) database was analyzed for expression level, mutation status, potential biological functions, and prognostic performance of core circadian clock genes in COAD. Their correlations with immune infiltration and TMB/MSI score were analyzed by Spearman's correlation analysis. Pearson's correlation analysis was performed to analyze their associations with drug sensitivity. Lasso Cox regression analysis was performed to construct a prognosis signature. Moreover, an mRNA-miRNA-lncRNA regulatory axis was also detected by ceRNA network.

**Results:**

In COAD tissues, the mRNA levels of *CLOCK*, *CRY1*, and *NR1D1* were increased, while the mRNA levels of *ARNTL*, *CRY2*, *PER1*, *PER3*, and *RORA* were decreased. We also summarized the relative genetic mutation variation landscape. GO and KEGG pathway analyses demonstrated that these circadian clock genes were primarily correlated with the regulation of circadian rhythms and glucocorticoid receptor signaling pathways. COAD patients with high *CRY2*, *NR1D1*, and *PER2* expression had worse prognosis. A prognostic model constructed based on the 9 core circadian clock genes predicted the COAD patients' overall survival with medium to high accuracy. A significant association between prognostic circadian clock genes and immune cell infiltration was found. Moreover, the lncRNA *KCNQ1OT1*/*hsa-miRNA-32-5p*/*PER2*/*CRY2* regulatory axis in COAD was also detected through a mRNA-miRNA-lncRNA network.

**Conclusion:**

Our results identified *CRY2*, *NR1D1*, and *PER2* as potential prognostic biomarkers for COAD patients and correlated their expression with immune cell infiltration. The lncRNA *KCNQ1OT1*/*hsa-miRNA-32-5p*/*PER2*/*CRY2* regulatory axis was detected in COAD and might play a vital role in the occurrence and progression of COAD.

## 1. Introduction

Colon adenocarcinoma (COAD) has become a severe health burden that causes over half a million cancer-related deaths annually [1]. COAD is the most common type of colon tumor. In many developing countries, patients with COAD are usually diagnosed at an advanced stage due to the lack of obvious symptoms at an early stage, related screening projects, and screening instruments [2]. The quality of life of advanced COAD patients is very low due to severe digestive symptoms [3]. Although surgical treatment, targeted treatment, chemotherapy, and immunotherapy have been flexibly utilized in the treatment of COAD, the prognosis of COAD patients is still disappointing [1]. It has been reported that the overall five-year survival rate of COAD patients is less than 40%, mainly due to postoperative recurrence and metastasis [4, 5]. Recently, aberrant expression of genes in tumor tissues and their potential applications in clinical practice have been a focus of clinicians. Thus, exploring potential gene signatures for therapeutic and prognostic assessments of COAD is significant clinically.

Physiological circadian rhythms are vital for various biological functions, such as metabolism, regeneration, immunology, and endocrinology [6]. All human tissues are modulated by the circadian clock through extremely complicated processes. The molecular circadian clock consists of a core clock gene loop, which mainly includes *CLOCK*, *ARNTL*, *PER1*, *PER2*, *PER3*, *CRY1*, *CRY2*, *NR1D1*, and *RORA* [6, 7]. In recent years, an increasing number of studies have attempted to explore the association between the circadian clock and cancer. Previous studies have found that disruption of normal rhythms and dysfunction of core clock gene loops contribute to the occurrence and development of many cancer types and influence the functions of the tumor immunity cycle [8–11]. For cancer therapy, previous studies revealed that cancer chronotherapy, i.e., timing chemotherapy administration based on circadian rhythms, might reduce the toxicity of drugs [12]. Some studies based on animal models have reported that drugs targeting biological clock targets, such as *RORγ* synthetic agonists and *RORα* synthetic agonists, could promote the activation of anticancer immunity [13, 14]. Nevertheless, the role of the circadian clock in prognosis evaluation and its clinical significance in COAD are rarely discussed.

With the rapid development of bioinformatics databases and the enrichment of genomic data, comprehensive analysis of the role of the circadian clock in COAD has become feasible. The present study explores the expression profiles, prognostic value, and related regulatory axis of the circadian clock in COAD, which may offer novel insights into prognostic biomarkers and biological clock targets for COAD.

## 2. Materials and Methods

### 2.1. Datasets

On May 20, 2021, COAD patient genomic data were downloaded from The Cancer Genome Atlas (TCGA) database. TCGA-COAD dataset (*N* = 455) was selected to perform the analyses. The corresponding clinical data, mainly including sex, age, tumor grade, and survival data, were also obtained from TCGA database on May 20, 2021. Furthermore, the copy number variation (CNV) and somatic mutation data in COAD were also extracted from TCGA database using UCSC Xena (https://xena.ucsc.edu/), an easy-to-use web portal for data files derived from TCGA. Setting the *P* value cutoff as 0.05, statistical analyses were conducted using R software V4.0.3. Before subsequent processing, the expression data were standardized to transcripts per kilobase million (TPM). To further analyze the expression of circadian clock genes, another gene expression profile of COAD was obtained from Oncomine (https://www.oncomine.org) [15], a cancer microarray database and integrated data-mining platform.

### 2.2. Identification of Differentially Expressed Core Circadian Clock Genes

A total of 9 genes were selected to perform further analysis, which included *CLOCK*, *ARNTL*, *PER1*, *PER2*, *PER3*, *CRY1*, *CRY2*, *NR1D1*, and *RORA* [6, 7]. The profiles of 24-hour oscillations of the core circadian clock genes were downloaded from CircaDB (https://circadb.hogeneschlab.org) [16]. For each tissue that was cyclic ordering by periodic structure (CYCLOPS) ordered, cosinor regression was used to test if individual genes are rhythmic or not [17]. In the 24-hour oscillation plots, the *x*-axis represented the CYCLOPS-estimated sample phase in radians (from 0 to 2𝝿). The *y*-axis represented the expression level (TPM). The time 𝝿 represented E-box phase (i.e., time of peak expression of E-box target genes *NR1D1*, *NR1D2*, and *PER3*). The datasets concerning the expression levels of the 9 core circadian clock genes were also excavated from the Oncomine (https://www.oncomine.org) database [15]. To further verify the expression level of the 9 core circadian clock genes, we also analyzed their expression in TCGA-COAD dataset. Student's *t*-test was utilized to compare the transcriptional levels of the core circadian clock genes in COAD tissues with those in normal colon tissues using the “reshape2” and “limma” packages in R software.

### 2.3. PPI Network and Mutation Analysis of Core Circadian Clock Genes

To study the correlation between each member of the 9 core circadian clock genes, we constructed a PPI network with the Search Tool for the Retrieval of Interacting Genes (STRING, https://string-db.org/), a database of predicted functional associations between genes and proteins [18]. Using R software, the overall mutation status of core circadian clock genes in COAD, including mutation categories and mutation frequencies of *CLOCK*, *ARNTL*, *PER1*, *PER2*, *PER3*, *CRY1*, *CRY2*, *NR1D1*, and *RORA* as well as their waterfall plot, were constructed using the “maftools” package. The locations of variants of the 9 genes on 23 chromosomes were shown using the “RCircos” package.

### 2.4. Functional Enrichment Analysis for Core Circadian Clock Genes

To identify the potential molecular mechanisms and biological functions of core circadian clock genes in COAD, functional enrichment analysis, including gene ontology (GO) analysis and Kyoto Encyclopedia of Genes and Genomes (KEGG) analysis, was performed using the “ggplot2” package in R software. In addition, GO analysis included biological process (BP), cellular component (CC), and molecular function (MF) analyses.

### 2.5. Construction of a Prognostic Model for Core Circadian Clock Genes

To assess the potential prognostic value of core circadian clock genes, Kaplan–Meier plots, including overall survival (OS) plots, disease-free survival (DFS) plots, disease-specific survival (DSS) plots, and progression-free survival (PFS) plots, were drawn. The hazard ratio (HR) and its 95% confidence interval (CI) are also listed in the plots. Based on the 9 core circadian clock genes, a LASSO Cox regression model was used to predict the influence of the expression of core circadian clock genes on the prognosis of COAD patients. According to the median risk score, patients were divided into the low- and high-risk groups, and the OS curves of the two groups were compared. The predictive accuracy of this prognostic model was assessed by time ROC analysis. Moreover, core circadian clock genes with potential prognostic value were selected for further analyses.

### 2.6. Immune Infiltration, TMB, MSI, and Drug Sensitivity Analyses

TIMER (https://cistrome.shinyapps.io/timer/) is a bioinformatics platform that is aimed at visualizing the association between immune cell infiltration and target gene expression in cancer cells [19]. The “Gene” module in TIMER was utilized to determine the correlation between the expression of prognostic circadian clock genes and the immune infiltration levels of B cells, CD8+ T cells, CD4+ T cells, neutrophils, dendritic cells, and macrophages. Based on the “ggstatsplot” package in R, tumor mutation burden (TMB) and microsatellite instability (MSI) analyses were used to assess the relationship between the expression of prognostic circadian clock genes and TMB and MSI scores, respectively. Spearman's correlation test was selected for the above analysis with a *P* value cutoff of 0.05. Moreover, to study the effect of prognostic circadian clock gene expression on drug resistance, drug sensitivity data in the Genomics of Drug Sensitivity in Cancer (GDSC) database were extracted.

### 2.7. Construction of the mRNA-miRNA-lncRNA Network

An mRNA-miRNA-lncRNA network was constructed to determine the potential regulatory axis of prognostic circadian clock genes in COAD. The miRNA targets binding to prognostic circadian clock genes were identified by the miRTarBase (http://mirtarbase.mbc.nctu.edu.tw/) [20] and starBase (https://starbase.sysu.edu.cn/index.php) [21] databases. miRTarBase and starBase are bioinformatics tools that can be used to predict miRNA targets of mRNAs. The mRNA-miRNA regulatory network was constructed using Cytoscape software. The Cytohubba plug-in was used to select the most significantly connected miRNAs. Next, according to the selected miRNAs, the lncRNA targets interacting with miRNAs were extracted from starBase (https://starbase.sysu.edu.cn/index.php) and lncBase (https://diana.e-ce.uth.gr/lncbasev3) [22], and the associations between the miRNAs and the lncRNAs were visualized. starBase and lncBase are bioinformatics tools that can be used to predict lncRNA targets of miRNAs. To further confirm the significant role of miRNAs and lncRNAs, we also evaluated the expression levels and prognostic performances of the targeted miRNAs and lncRNAs using TCGA-COAD dataset.

## 3. Results

### 3.1. Work Flow of the Current Study

**Figure 1:**
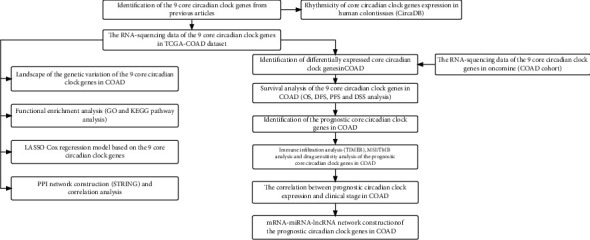
Flow chart of the current study.

Work flow of the present study is shown in Figure 1. Firstly, the core circadian clock genes in human were identified based on the previous studies [6, 7]. To reveal their rhythmicity in human colon tissues, the 24-hour oscillations of these genes were listed based on the expression profiles in CircaDB. Next, to find out the differentially expressed genes, we downloaded the RNA-sequencing data in Oncomine and TCGA-COAD dataset, respectively. The datasets in Oncomine were from the previous publications. Through comparing the expression profiles in the two databases, the expression status of core circadian clock genes in COAD was more trustworthy. And then, based on clinical data in TCGA-COAD database, the potential prognostic value of these genes in COAD was also analyzed by Kaplan-Meier methods, which included OS, DFS, PFS, and DSS. After screening out the prognostic circadian clock genes in COAD, we were also interested on the correlation between prognostic circadian clock genes expression and immune cell infiltration; these results might assist researchers to explore potential immunotherapeutic target for COAD patients. Immune infiltration analysis was conducted by TIMER. MSI and TMB could predict the efficacy of cancer immunotherapy. In theory, cancer patients with high TMB/MSI score might get better therapeutic effect. Thus, we detected the influence of prognostic circadian clock genes expression on TMB/MSI score. Meanwhile, the association between gene expression and drug sensitivity of colon tissues was also analyzed. To explore potential genes that might influence the development of COAD, we performed the subgroup analysis to show the correlation between gene expression and clinical stage. In addition, we attempted to explore a potential mRNA-miRNA-lncRNA regulatory axis concerning these prognostic circadian clock genes in COAD. To comprehensively investigate these 9 genes in COAD, the following analyses were performed. Mutation analysis helped readers to intuitively observe the overall mutation status of circadian clock genes in COAD. As the core genes in circadian clock loop, the potential biological functions and molecular mechanisms of these 9 genes were investigated by functional enrichment analysis. These results would provide novel insights on the functional studies of circadian clock genes. And in recent years, machine learning and big data technique were used to predict prognosis. Thus, LASSO Cox regression model based on the core circadian clock genes was constructed. Finally, the PPI network was constructed to visualize the correlation between each member of the 9 core circadian clock genes. Correlation analysis was performed to show the coexpression among these 9 genes in COAD, thus revealing synergistic interaction on expression among these genes.

### 3.2. Expression of Core Circadian Clock Genes in COAD and Normal Colon Tissues

**Figure 2:**
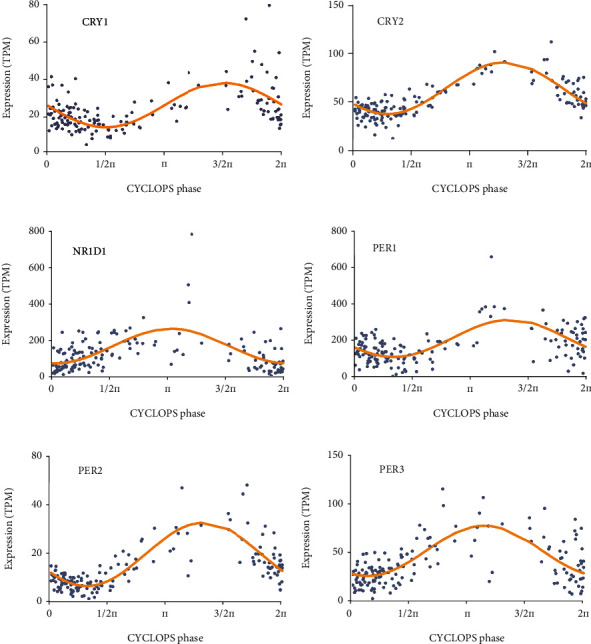
The 24-hour oscillations of core circadian clock genes in human colon tissues (CircaDB), including *CRY1* (a), *CRY2* (b), *NR1D1* (c), *PER1* (d), *PER2* (e), and *PER3* (f).

**Figure 3:**
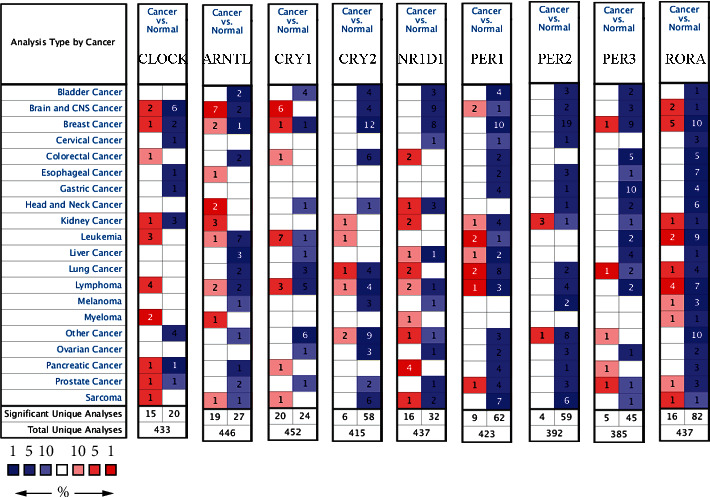
The transcription levels of core circadian clock genes in different cancer types (Oncomine). The numbers in red boxes represent the numbers of datasets that indicate upregulated expression of target genes in corresponding cancer types, while the numbers in blue boxes represent the numbers of datasets that reveal downregulated expression of target genes in corresponding cancer types.

**Figure 4:**
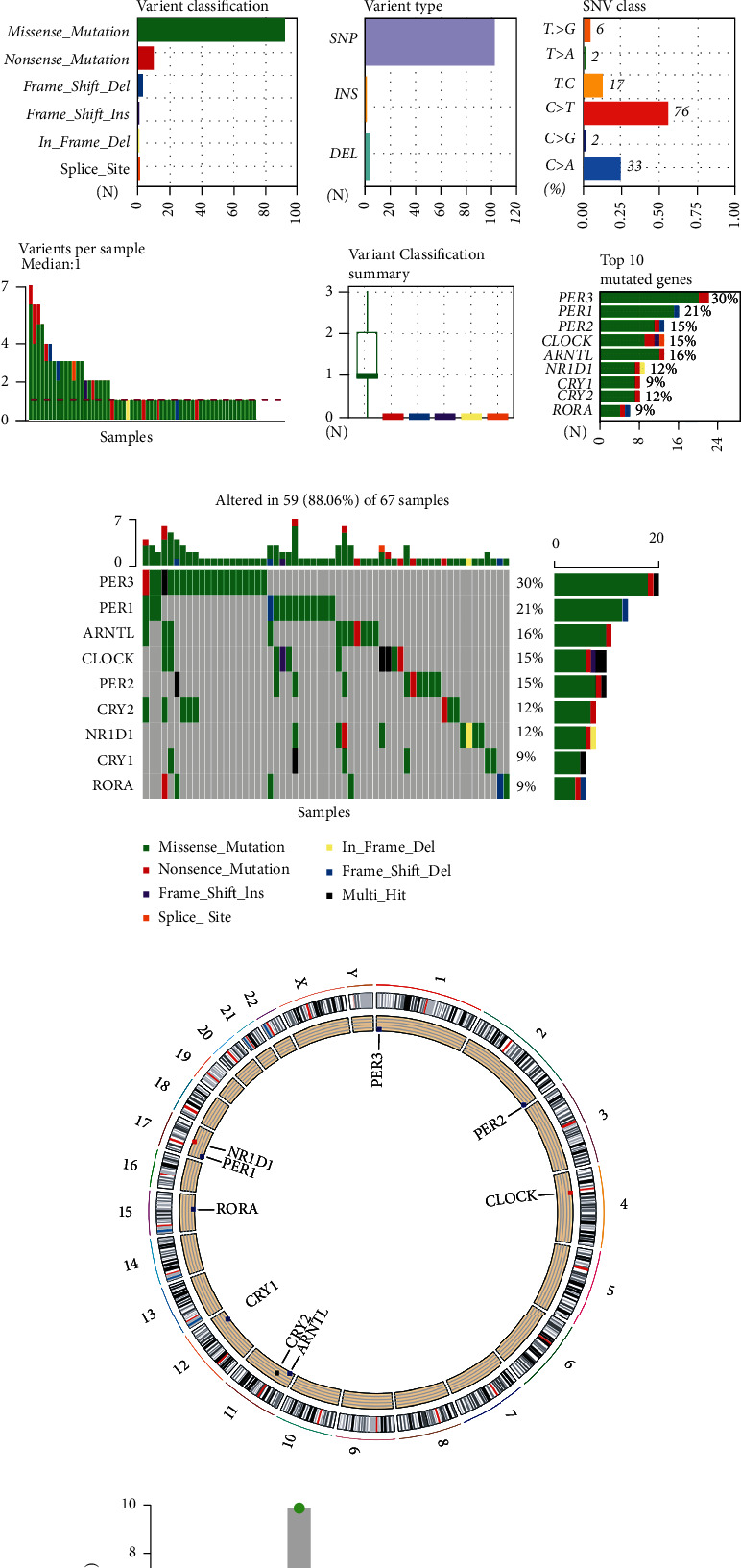
Landscape of genetic variation and expression of core circadian clock genes in COAD. (a) The mRNA levels of core circadian clock genes in COAD and normal colon tissues. (b, c) The mutation frequency and classification of core circadian clock genes in COAD. (d) The location of CNV alteration of core circadian clock genes on 23 chromosomes in COAD. (e) The CNV variation frequency of core circadian clock genes in COAD. The height of the column represents the alteration frequency. CNV: copy number variation; COAD: colon adenocarcinoma. ^∗∗∗^*P* < 0.001.

The 24-hour oscillations of *CRY1* (Figure 2(a)), *CRY2* (Figure 2(b)), *NR1D1* (Figure 2(c)), *PER1* (Figure 2(d)), *PER2* (Figure 2(e)), and *PER3* (Figure 2(f)) in human colon tissues were visualized according to the expression profiles in CircaDB. However, there were no *CLOCK*, *ARNTL*, or *RORA* expression level data in CircaDB. Based on the Oncomine database, the number of datasets that contained the expression profiles of *CLOCK*, *ARNTL*, *PER1*, *PER2*, *PER3*, *CRY1*, *CRY2*, *NR1D1*, and *RORA* is shown in Figure 3. In colorectal cancer, the transcriptional levels of *CLOCK*, *CRY1*, and *NR1D1* were upregulated in 1, 1, and 2 datasets, respectively. The transcriptional levels of *ARNTL*, *CRY2*, *PER1*, *PER3*, and *RORA* were downregulated in 2, 6, 2, 5, and 5 datasets, respectively. Using TCGA-COAD dataset, the mRNA levels of the 9 core circadian clock genes in TCGA-COAD tissues and the corresponding mRNA levels in normal colon tissues were identified and are shown in Figure 4(a). Specifically, the mRNA levels of *CLOCK*, *CRY1*, and *NR1D1* were significantly higher in COAD tissues, while the mRNA levels of *ARNTL*, *CRY2*, *PER1*, *PER3*, and *RORA* were significantly decreased in COAD tissues (all *P* values < 0.001). At the transcriptional level, these core circadian clock genes were greatly altered in COAD tissues. Moreover, a protein–protein interaction (PPI) network was constructed to illustrate the associations among the 9 core circadian clock genes (Supplementary Figure 1(a)). Correlation analysis of core circadian clock genes showed significant positive correlations with each other apart from *NR1D1* in TCGA-COAD dataset, suggesting that these core circadian clock genes had synergistic interactions on expression (Supplementary Figure 1(b)).

### 3.3. Landscape of the Genetic Variation of Core Circadian Clock Genes in COAD Tissues

Next, we summarized the somatic variations (SNVs) and copy number variations (CNVs) of the 9 circadian clock genes in COAD. As shown in Figures 4(b) and 4(c), genetic alterations were detected in 59 (88.06%) of 67 COAD samples. The categories of genetic mutations included missense mutation, nonsense mutation, frame-shift insertion, frame-shift deletion, in-frame deletion, splice-site variation, and multi-hit mutation. The most common variant classification was missense mutation. In addition, SNPs were the most common variant type, and C>T ranked as the top SNV class. Of these genes, *PER3* was the gene with the highest mutation frequency. The locations of the CNV variations on chromosomes are also presented in Figure 4(d). The CNV variation frequencies of the 9 core circadian clock genes in COAD are also summarized in Figure 4(e). Copy number amplification was shown in *NR1D1* and *CLOCK*, while copy number deletion was shown in *PER3*, *PER2*, *RORA*, *ARNTL*, *CRY1*, and *PER1*.

### 3.4. Functional Enrichment Analysis of Core Circadian Clock Genes in COAD Tissues

**Figure 5:**
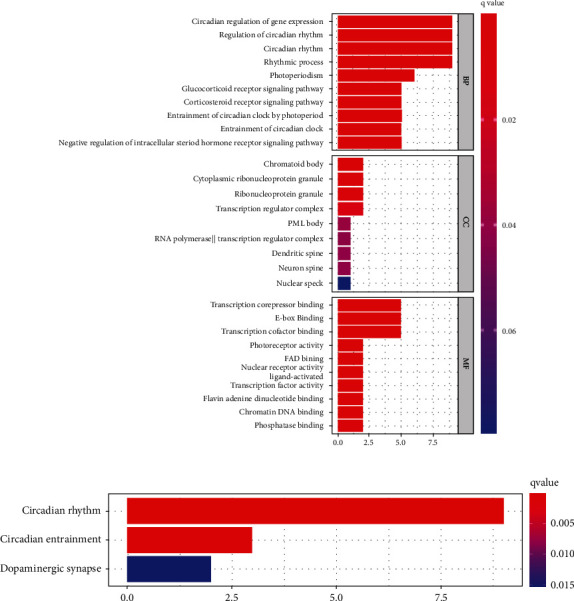
Functional enrichment analysis of core circadian clock genes in COAD. GO analyses (a), including BP analysis, CC analysis and MF analysis, and KEGG pathway analysis (b), were performed. COAD: colon adenocarcinoma; GO: Gene Ontology; BP: biological process; CC: cellular component; MF: molecular function; KEGG: Kyoto Encyclopedia of Genes and Genomes; HR: hazard ratio.

To clarify the potential biological functions and molecular mechanisms of core circadian clock genes in COAD, GO and KEGG pathway analyses were performed (Figure 5). GO analysis, including BP, CC, and MF analyses, illustrated that these 9 genes mainly participated in the regulation of circadian rhythm, circadian regulation of gene expression, rhythmic process, glucocorticoid receptor signaling pathway, corticosteroid receptor signaling pathway, transcription corepressor binding, and E-box binding (Figure 5(a)). KEGG pathway analysis revealed that the 9 core circadian clock genes were mainly involved in the regulation of circadian rhythm, circadian entrainment and dopaminergic synapses (Figure 5(b)).

### 3.5. Prognostic Model Construction of Core Circadian Clock Genes in COAD Tissues

**Figure 6:**
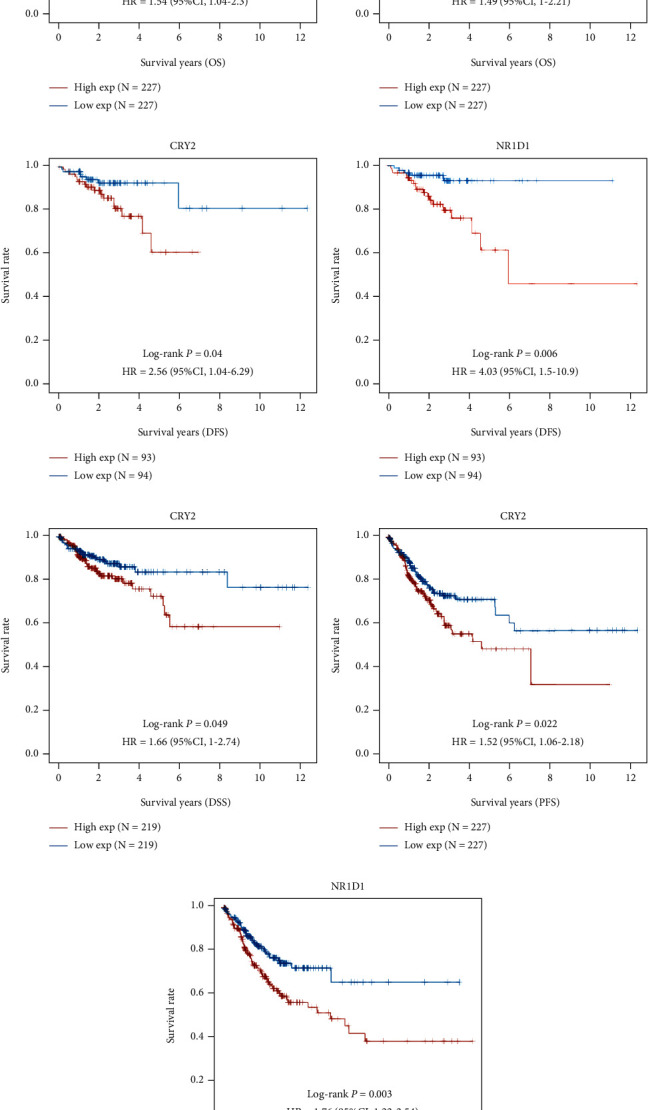
The prognostic value of core circadian clock genes in COAD. OS (a), DFS (c), DSS (e), and PFS (f) of COAD patients with high/low *CRY2* expression. OS (b) of COAD patients with high/low *PER2* expression. DFS (d) and PFS (g) of COAD patients with high/low *NR1D1* expression. COAD: colon adenocarcinoma; OS: overall survival; DFS: disease-free survival; DSS: disease-specific survival; PFS: progression-free survival.

**Figure 7:**
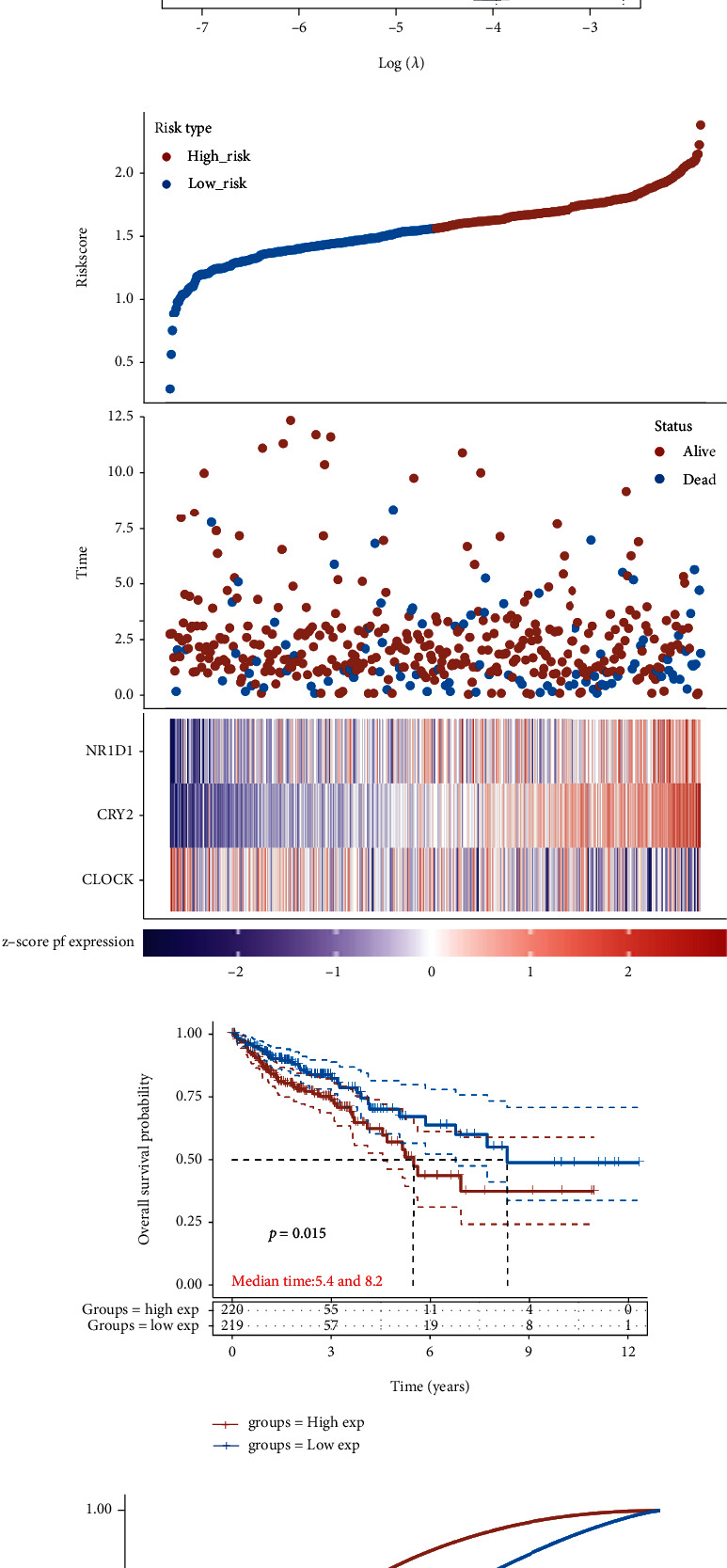
Prognostic PRG model of core circadian clock genes in COAD. (a, b) Lasso Cox regression analysis of core circadian clock genes in COAD. (c) Distribution of risk score, survival status, and the expression of the genes that take part in the formation of the risk score. (d, e) Overall survival analysis for patients at high/low risk and the ROC curve for measuring the predictive value. COAD: colon adenocarcinoma.

Kaplan–Meier plots, including OS, DFS, PFS, and DSS plots, were used to assess the potential prognostic value of the 9 core circadian clock genes. The *P* values and their HRs with 95% CIs are shown in Table 1. COAD patients with high *CRY2* expression showed worse OS (Figure 6(a)) (*P* value = 0.032, HR with 95% CI = 1.54 (1.04-2.30)), DFS (Figure 6(c)) (*P* value = 0.040, HR with 95%CI = 2.56 (1.04-6.29)), DSS (Figure 6(e)) (*P* value = 0.049, HR with 95%CI = 1.66 (1.00-2.74)), and PFS (Figure 6(f)) (*P* value = 0.022, HR with 95%CI = 1.52 (1.06-2.18)). COAD patients with high NR1D1 expression showed worse DFS (Figure 6(d)) (*P* value = 0.006, HR with 95% CI = 4.03 (1.50-10.90)) and PFS (Figure 6(g)) (*P* value = 0.003, HR with 95%CI = 1.76 (1.22-2.54)). In addition, COAD patients with high *PER2* expression showed worse OS (Figure 6(b)) (*P* value = 0.047, HR with 95%CI = 1.49 (1.00-2.21)). The above results demonstrated that *CRY2*, *NR1D1* and *PER2* might be of great significance in the prognostic evaluation of COAD patients. Moreover, a prognostic model including the 9 core circadian clock genes was constructed by Lasso Cox regression analysis (Figure 7(a)–7(b)): risk score = (−0.1332)∗*CLOCK* + (0.4144)∗*CRY*2 + (0.0934)∗*NR*1*D*1. According to the risk score, patients were divided into the high- and low-risk groups. The score distribution, survival status, and expression levels of *PER2*, *NR1D1*, and *CLOCK* are shown in Figure 7(c). When the risk score increased, the risk of death of COAD patients increased, while the survival time decreased (Figure 7(c)). Compared with the low-risk group, COAD patients with high risk showed a worse OS (median survival time = 5.4 vs. 8.2 years, *P* value = 0.015, Figure 7(d)). Furthermore, the AUCs of the 5- and 10-year ROC curves were 0.604 (95% CI: 0.512-0.697) and 0.696 (95% CI: 0.536-0.856), respectively (Figure 7(e)), which showed medium to high accuracy.

### 3.6. Immune Infiltration Analysis for Prognostic Circadian Clock Genes in COAD Tissues

**Figure 8:**
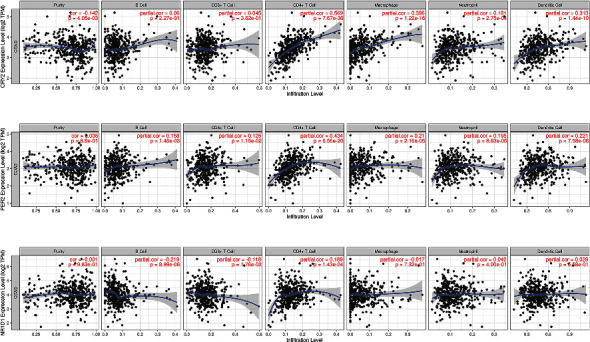
The correlations between *CRY2*, *PER2* and *NR1D1* and immune cell infiltration in COAD (TIMER). (a) The correlations between *CRY2* and the abundance of six types of tumor-infiltrating cells. (b) The correlations between *PER2* and the abundance of six types of tumor-infiltrating cells. (c) The correlations between *NR1D1* and the abundance of six types of tumor-infiltrating cells. COAD: colon adenocarcinoma; cor: correlation coefficient.

The above study revealed that *CRY2*, *NR1D1*, and *PER2* were potential biomarkers for COAD. We then selected *CRY2*, *NR1D1*, and *PER2* for further analysis because of their prognostic performance. The association between the infiltration level of immune cells and the expression level of prognostic circadian clock genes in COAD was analyzed. As shown in Figure 8(a), the expression level of *CRY2* was positively correlated with the infiltration levels of CD4+ T cells (cor = 0.569, *P* value = 7.67*E* − 36), macrophages (cor = 0.396, *P* value = 1.22*E* − 16), neutrophils (cor = 0.181, *P* value = 2.75*E* − 04), and dendritic cells (cor = 0.313, *P* value = 1.44*E* − 10). The expression level of *PER2* was positively associated with the infiltration levels of B cells (cor = 0.158, *P* value = 1.45*E* − 03), CD8+ T cells (cor = 0.125, *P* value = 1.15*E* − 02), CD4+ T cells (cor = 0.434, *P* value = 6.56*E* − 20), macrophages (cor = 0.210, *P* value = 2.16*E* − 05), neutrophils (cor = 0.195, *P* value = 8.63*E* − 05), and dendritic cells (cor = 0.221, *P* value = 7.58*E* − 06) (Figure 8(b)). In addition, the expression level of *NR1D1* was positively associated with the infiltration level of CD4+ T cells (cor = 0.189, *P* value = 1.43*E* − 04) and negatively associated with the infiltration levels of B cells (cor = −0.219, *P* value = 8.99*E* − 06) and CD8+ T cells (cor = −0.118, *P* value = 1.76*E* − 02) (Figure 8(c)). We were also interested in the correlation between the common immune biomarkers and the expression levels of prognostic circadian clock genes in COAD. As shown in Table 2, *CRY2* expression was positively correlated with most of the gene markers on immune cells. However, *PER2* and *NR1D1* expression levels were significantly correlated with only 24 and 29 kinds of gene markers, respectively. These results revealed that *CRY2* might be a potential immunotherapeutic target for COAD patients.

### 3.7. TMB, MSI, and Drug Sensitivity Analyses of Core Circadian Clock Genes in COAD Tissues

**Figure 9:**
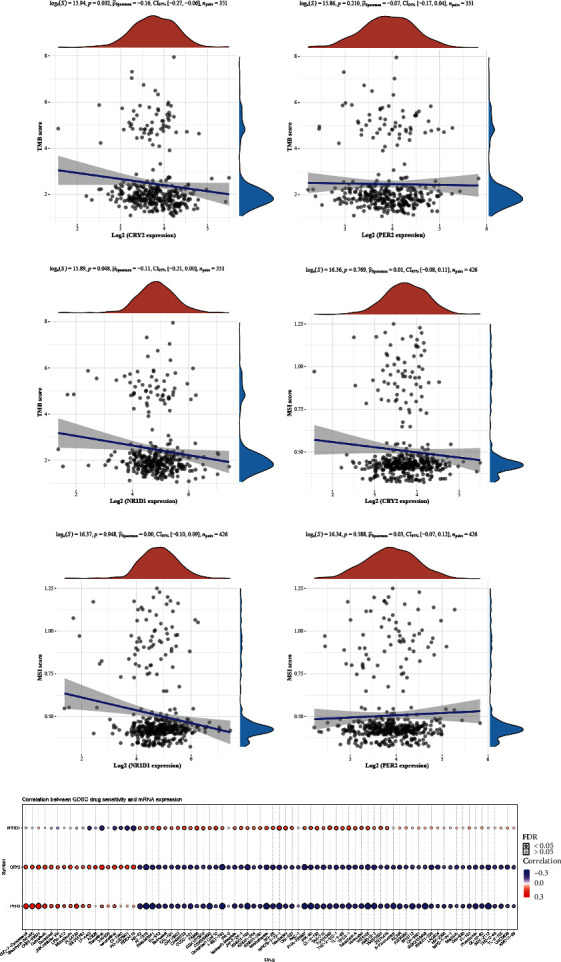
TMB, MSI, and drug sensitivity analyses of core circadian clock genes in COAD. (a–c) The correlations between *CRY2*, *PER2*, *NR1D1*, and TMB. (d–f) The correlations between *CRY2*, *NR1D1*, *PER2*, and MSI. (g) The correlations between the prognostic circadian clock genes and drug sensitivity based on the GDSC database. COAD: colon adenocarcinoma; TMB: tumor mutation burden; MSI: microsatellite instability; GDSC: Genomics of Drug Sensitivity in Cancer.

Tumor mutation burden (TMB) and microsatellite instability (MSI) are of great significance in predicting the efficacy of cancer immunotherapy. The above results demonstrated that the prognostic circadian clock genes were associated with immune cell infiltration. Therefore, we conducted TMB and MSI analyses to assess the potential clinical applications of *CRY2*, *NR1D1*, and *PER2* in COAD immunotherapy. In TMB analysis, negative correlations were detected between TMB and *CRY2* expression (Figure 9(a), *P* value = 0.002) and *NR1D1* expression (Figure 9(c), *P* value = 0.048), while no significant correlation was found between TMB and *PER2* expression (Figure 9(b), *P* value = 0.210). In MSI analysis, unexpectedly, no significant associations between MSI and *CRY2* expression (Figure 9(d), *P* value = 0.769), *NR1D1* expression (Figure 9(e), *P* value = 0.948), or *PER2* expression (Figure 9(f), *P* value = 0.588) were detected. Next, drug sensitivity analysis was performed to detect the potential functions of prognostic circadian clock genes in drug screening. Based on data from the GDSC database, high *NR1D1* expression in COAD tissues was mainly correlated with drug resistance to 38 kinds of drugs and drug sensitivity to 6 kinds of drugs. High *PER2* expression was primarily associated with drug sensitivity in 58 kinds of drugs and drug resistance in 13 kinds of drugs, while high *CRY2* expression was mainly associated with drug sensitivity in 60 kinds of drugs and drug resistance in 18 kinds of drugs (Figure 9(g)), indicating that *PER2* and *CRY2* were potential biomarkers for drug screening in COAD.

### 3.8. Construction of a mRNA-miRNA-LncRNA Network

**Figure 10:**
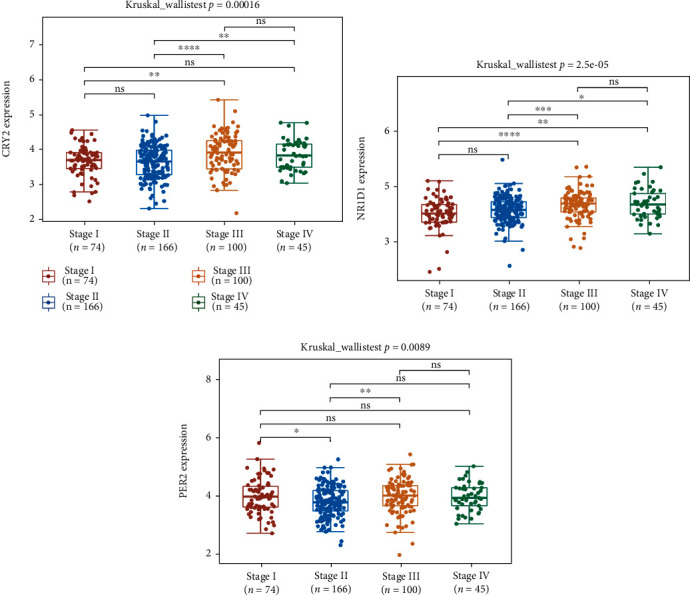
Correlations between the prognostic circadian clock genes and clinical stage in COAD. Correlations between clinical stage and *CRY2* (a), *NR1D1* (b), and *PER2* (c) in COAD. COAD: colon adenocarcinoma. ^∗^*P* < 0.05,  ^∗∗^*P* < 0.01, and^∗∗∗^*P* < 0.001.

**Figure 11:**
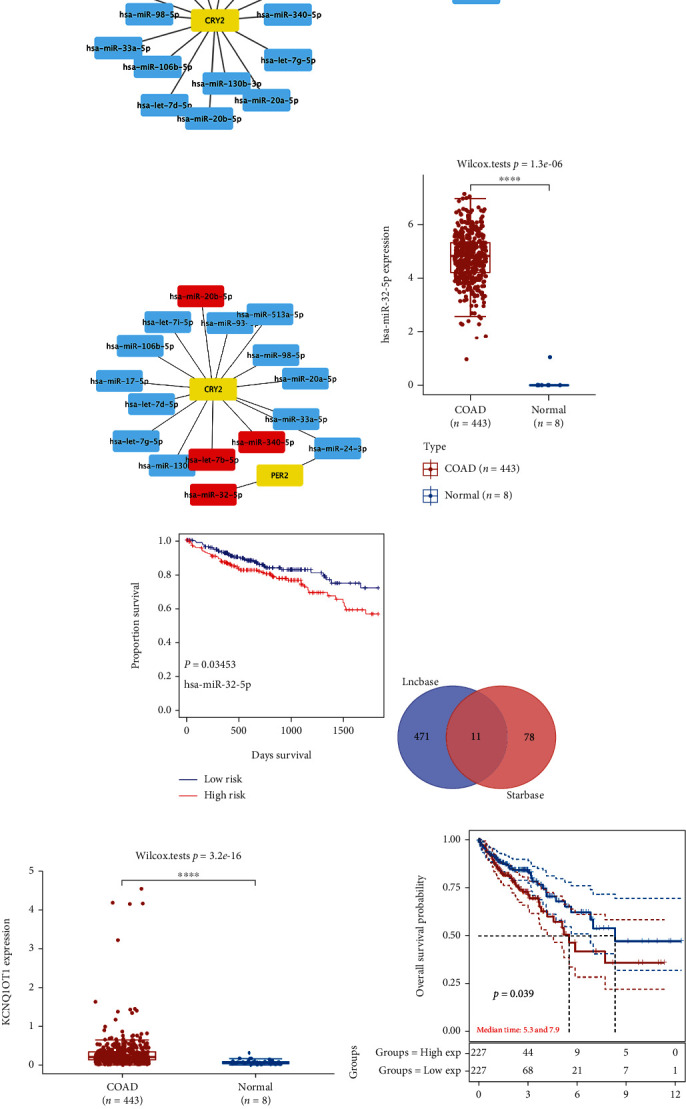
Construction of an mRNA-miRNA-lncRNA network. (a) Results of miRNA targets predicted by miRTarBase and starBase. (b) Target miRNAs with dysexpression. The expression (c) and prognostic value (d) of *miR-32-5p* in COAD. (e) Results of lncRNA targets predicted by lncBase and starBase. The expression (f) and prognostic value (g) of lncRNA *KCNQ1OT1* in COAD. COAD: colon adenocarcinoma.

The relationship between clinical stage and the expression of prognostic circadian clock genes was analyzed. As shown in Figure 10, the expression levels of *CRY2* (Figure 10(a), *P* value = 0.00016), *NR1D1* (Figure 10(b), *P* value = 2.5*E* − 05), and *PER2* (Figure 10(c), *P* value = 0.0089) were associated with the clinical stage of COAD patients, which demonstrated that these 3 genes might be involved in the development of COAD. However, for potential applications in drug screening and prognostic performance, *PER2* and *CRY2* were selected to construct an mRNA-miRNA-lncRNA network. The miRNAs targeting *PER2* and *CRY2* identified by starBase and miRTarBase are shown in Figure 11(a). Among these miRNAs, *hsa-miRNA-32-5p*, *hsa-miRNA-340-5p*, *hsa-miRNA-20b-5p*, and *hsa-let-7b-5p* were identified as the top highly connected miRNAs (Figure 11(b)), which were upregulated or downregulated in COAD tissues. We then detected the expression profiles and prognostic value of these four genes. The results showed that only *hsa-miRNA-32-5p* was differentially expressed in tumors and was significantly associated with patient prognosis. More specifically, the expression of *hsa-miRNA-32-5p* was upregulated in COAD tissues compared with normal colon tissues (*P* value = 1.3*E* − 06, Figure 11(c)). COAD patients with high *hsa-miRNA-32-5p* levels showed worse OS (*P* value = 0.03453, Figure 11(d)). Thus, hsa-miRNA-32-5p was selected as the most promising miRNA target of *PER2* and *CRY2*. Next, we further detected the upstream lncRNA targets using lncBase and starBase. According to the data from the above databases, a total of 11 lncRNA targets were identified (Figure 11(e)), including *NDUFA6-AS1*, *SNHG14*, *CTBP1-AS2*, *LINC01128*, *INTS6-AS1*, *XIST*, *LINC00365*, *KCNQ1OT1*, *OIP5-AS1*, *DAPK1-IT1*, and *PITPNA-AS1*. The expression levels of the above lncRNA targets in COAD tissues and normal colon tissues were analyzed. Similarly, the results showed that only lncRNA *KCNQ1OT1* was differentially expressed in tumors and was significantly associated with patient prognosis. The data demonstrated upregulation of the lncRNA *KCNQ1OT1* in COAD tissues compared with normal colon tissues (*P* value = 3.2*E* − 16, Figure 11(f)). COAD patients with high *KCNQ1OT1* expression had a worse OS than those with low *KCNQ1OT1* expression (median survival time = 5.3 years vs. 7.9 years, *P* value = 0.039, Figure 11(g)). Thus, the lncRNA *KCNQ1OT1*/*hsa-miRNA-32-5p*/*PER2/CRY2* regulatory axis might influence the occurrence and progression of COAD.

## 4. Discussion

In 1971, scientists first found three variations in one gene on the X chromosome of *Drosophila melanogaster*; one caused physiological response and behavior to become completely asynchronous with the circadian clock, and the other two caused the circadian clock to change to 19 and 28 hours, respectively [23]. By analyzing the entire X chromosome, researchers identified the location of the gene and named the gene *PER*. After this groundbreaking study, many studies attempted to expand the circadian clock gene family and determine the potential roles of circadian clock genes in the occurrence and development of disease and their potential applications in disease diagnosis, treatment and prevention. In the past 20 years, the circadian clock has been studied at the genetic level. The genes that participated in the construction of the circadian clock loop mainly included *CLOCK*, *ARNTL*, *PER1*, *PER2*, *PER3*, *CRY1*, *CRY2*, *NR1D1*, and *RORA* [6, 7]. Previous studies reported that dysfunction and abnormal expression of these genes were involved in the occurrence and progression of various human cancer types [8, 9, 24, 25]. Some studies have focused on the potential of core circadian clock genes to be cancer biomarkers [26–28]. Nevertheless, the specific functions of core circadian clock genes in the prognosis and therapy of COAD are rarely discussed. Thus, we adopted bioinformatics methods to illustrate the potential role of core circadian clock genes in COAD.

To a certain extent, the expression of core circadian clock genes reflects the status of the circadian clock and influences its functions [29]. In mammals, interlocked transcriptional-translational feedback loops regulate the circadian clock. In normal tissues, the positive limb of this loop is composed of *CLOCK* and *ARNTL*, which induce the expression of *CRY1-2* and *PER1-3*. In turn, overexpressed *PER* and *CRY* proteins suppress their own transcription, thus forming the circadian rhythm [30, 31]. Compared to those in normal colon tissues, the transcriptional levels of *CLOCK*, *CRY1*, and *NR1D1* in COAD tissues were significantly higher, while the mRNA levels of *ARNTL*, *CRY2*, *PER1*, *PER3*, and *RORA* in COAD tissues were downregulated in the current study. The dysexpression of these genes in COAD tissues might be involved in the disruption of circadian rhythms, thus leading to the occurrence and progression of COAD.

Researchers are also interested in the potential prognostic value of core circadian clock genes in COAD. In the present study, COAD patients with high *CRY2* levels showed worse OS, DFS, DSS, and PFS than those with low *CRY2* levels. COAD patients with high NR1D1 expression had worse DFS and PFS. In addition, high PER2 expression predicted a worse OS for COAD patients. A previous study based on human colorectal cancer tissues indicated that *CRY2* expression was decreased and that high *CRY2* expression predicted worse OS, which supported the results of the current study [26]. Na et al. indicated that *NR1D1* was considered a biomarker predicting good prognosis in breast cancer patients [27]. *PER2* is considered a prognostic predictor in several human cancer types, including lung cancer, colorectal cancer, and gastric cancer [28, 32, 33]. Moreover, the overall prognostic performances of *CLOCK*, *PER1*, *PER3*, and *CRY1* were also determined in a previous meta-analysis [34].

Tumor-infiltrating immune cells play pivotal roles in eliminating cancer cells and hindering cancer progression. Exploring the association between core circadian clock genes and tumor-infiltrating immune cells is of significant importance. A previous study concluded the role of the circadian clock and its core genes in cancer immunity [35]. Xu et al. reported an M1-like proinflammatory phenotype of macrophages in mice with relatively disrupted *PER1* and *PER2* clock genes [36]. Cao et al. reported that *CRY* proteins regulated the process of autoimmunity, including B cell development, the BCR signaling pathway, and C1q expression, in CRY DKO (double knockout) mice [37]. However, more high-quality studies are needed to illustrate the mechanisms and specific processes of core circadian clock genes in cancer immunity. In addition, we detected the associations between the expression of core circadian clock genes in COAD and biomarkers in immune cells, which might assist researchers in determining potential immunotherapy targets for COAD. *RORγ* synthetic agonists could increase the expression levels of *IL-17A*, *IL-17F*, *GM-CSF*, and *IL-22*, thus boosting Th17 cells and Tregs to block immunosuppression [38]. The expression of *BMAL1*, *CLOCK*, *Rev-Erbα*, and *PER2* in intraperitoneal macrophages regulated the expression levels of F4/80 and CD11c in tumor tissues [39]. However, immunotherapy based on core circadian clock gene targets is still at the initial stage. Thus, more complementary studies aimed at core circadian clock genes are urgently needed.

GO and KEGG pathway analyses could predict the biological functions and molecular mechanisms of the target gene set. In our study, the main functions and mechanisms of the 9 core circadian clock genes were involved in the regulation of circadian rhythms and some signaling pathways. The corticosteroid receptor signaling pathway includes the glucocorticoid receptor signaling pathway and mineralocorticoid receptor signaling pathway. In a mouse model, So et al. identified glucocorticoid response elements in the molecular circadian clock and found that *PER2* was an integral component of the glucocorticoid regulatory pathway and was directly regulated by the glucocorticoid receptor [40]. Dickmeis et al. described multiple mechanisms by which molecular circadian clock genes regulate rhythms of glucocorticoid release and modulate glucocorticoid signaling [41]. For the mineralocorticoid receptor signaling pathway, circadian gene *CLOCK* signaling contributed to mineralocorticoid receptor-mediated cardiac inflammation and fibrosis [42]. The above findings revealed the significance of circadian clock genes in the corticosteroid receptor signaling pathway.

Through mRNA-miRNA-lncRNA network construction, the lncRNA *KCNQ1OT1*/*hsa-miRNA-32-5p*/*PER2*/*CRY2* regulatory axis was identified. Previous studies have reported that *miRNA-32-5p* regulates the progression of gastric cancer and epithelial-mesenchymal transition and metastasis in lung adenocarcinoma [43, 44]. However, studies concerning the role of *miRNA-32-5p* in COAD are lacking. The upstream lncRNA *KCNQ1OT1* was also detected and is considered a potential diagnostic biomarker for colon and rectal cancer and enhanced drug resistance in colon cancer cells [45, 46]. In the present study, we also studied the prognostic performance of *miRNA-32-5p* and lncRNA *KCNQ1OT1* and found that they were correlated with COAD patient prognosis. The above findings revealed that the lncRNA *KCNQ1OT1*/*hsa-miRNA-32-5p*/*PER2*/*CRY2* regulatory axis might be of significant importance in the occurrence and progression of COAD and may have potential applications in the diagnosis and treatment of COAD. Nevertheless, in vivo and vitro studies are urgently needed to clarify the role of this regulatory axis in COAD.

Some limitations still exist in the current study. First, our study and analysis were mainly performed at the transcriptional level, and the results may not be applicable in studies based on the protein level. Second, related fundamental and clinical studies that focus on the molecular mechanisms of circadian clock genes in colon or rectal cancer are rare. Third, the genetic background and etiology of COAD patients are influenced by many factors, such as patient race, sex, and age. Thus, more in-depth studies are necessary to validate the role of circadian clock genes in COAD.

## 5. Conclusion

In this study, a comprehensive bioinformatics analysis was used to identify the prognostic circadian clock gene signature in COAD, which included *CRY2*, *NR1D1*, and *PER2*. The lncRNA *KCNQ1OT1*/hsa-miRNA-32-5p/*PER2*/*CRY2* regulatory axis was identified and might be of great significance in the occurrence and progression of COAD.

## Figures and Tables

**Table 1:** Prognostic performance of core circadian genes in COAD based on TCGA cohort.

Genes	OS		DFS		DSS		PFS	
	*P* value	HR (95% CI)	*P* value	HR (95% CI)	*P* value	HR (95% CI)	*P* value	HR (95% CI)
*CLOCK*	0.295	0.81 (0.55-1.20)	0.246	0.61 (0.26-1.41)	0.155	0.70 (0.42-1.15)	0.444	0.87 (0.61-1.24)
*ARNTL*	0.745	1.07 (0.72-1.58)	0.837	1.09 (0.48-2.47)	0.695	1.10 (0.67-1.80)	0.934	1.02 (0.71-1.45)
*CRY1*	0.566	0.89 (0.60-1.31)	0.057	0.42 (0.17-1.03)	0.481	0.84 (0.51-1.37)	0.101	0.74 (0.52-1.06)
***CRY2***	0.032	1.54 (1.04-2.30)	0.040	2.56 (1.04-6.29)	0.049	1.66 (1.00-2.74)	0.022	1.52 (1.06-2.18)
***NR1D1***	0.211	1.29 (0.87-1.91)	0.006	4.03 (1.50-10.87)	0.404	1.24 (0.75-2.03)	0.003	1.76 (1.22-2.54)
*PER1*	0.220	1.28 (0.86-1.89)	0.884	1.06 (0.46-2.45)	0.424	1.22 (0.75-2.00)	0.063	1.40 (0.98-2.01)
***PER2***	0.047	1.49 (1.00-2.21)	0.069	2.29 (0.94-5.59)	0.077	1.57 (0.95-2.58)	0.065	1.40 (0.98-2.00)
*PER3*	0.885	1.03 (0.70-1.52)	0.561	1.28 (0.56-2.90)	0.790	0.94 (0.57-1.53)	0.671	1.08 (0.76-1.54)
*RORA*	0.895	0.97 (0.66-1.44)	0.817	0.91 (0.40-2.06)	0.948	0.98 (0.60-1.61)	0.735	1.06 (0.75-1.52)

**Table 2:** Correlations of *CRY2*, *NR1D1*, and *PER2* and gene markers on tumor-infiltrating cells in COAD (TIMER).

Description	Gene markers	COAD					
		CRY2		NR1D1		PER2	
		Cor	*P* value	Cor	*P* value	Cor	*P* value
CD8+ T cell	*CD8A*	0.118	∗	−0.143	∗∗	0.039	0.402
	*CD8B*	0.121	∗∗	−0.128	∗∗	0.031	0.511

T cell (general)	*CD3D*	0.102	∗	−0.158	∗∗∗	0.005	0.919
	*CD3E*	0.208	∗∗∗	−0.097	∗	0.087	0.062
	*CD2*	0.122	∗∗	−0.195	∗∗∗	0.054	0.245

B cell	*CD19*	0.194	∗∗∗	−0.031	0.513	0.130	∗∗
	*CD79A*	0.268	∗∗∗	−0.091	0.050	0.169	∗∗∗

Monocyte	*CD86*	0.228	∗∗∗	−0.153	∗∗	0.093	∗
	*CD115 (CSF1R)*	0.418	∗∗∗	−0.038	0.419	0.179	∗∗∗

TAM	*CCL2*	0.244	∗∗∗	−0.133	∗∗	0.026	0.575
	*CD68*	0.293	∗∗∗	0.038	0.417	0.105	∗
	*IL10*	0.132	∗∗	−0.195	∗∗∗	-0.008	0.868

M1 macrophage	*INOS (NOS2)*	-0.238	∗∗∗	0.006	0.898	-0.059	0.209
	*IRF5*	0.210	∗∗∗	0.152	∗∗	0.099	∗
	*COX2 (PTGS2)*	0.028	0.551	−0.011	0.823	0.087	0.062

M2 macrophage	*CD163*	0.314	∗∗∗	-0.090	0.053	0.145	∗∗
	*VSIG4*	0.255	∗∗∗	-0.111	∗	0.030	0.519
	*MS4A4A*	0.206	∗∗∗	-0.180	∗∗∗	0.047	0.313

Neutrophils	*CD66b (CEACAM8)*	-0.241	∗∗∗	-0.033	0.480	-0.174	∗∗∗
	*CD11b (ITGAM)*	0.336	∗∗∗	-0.010	0.837	0.122	∗∗
	*CCR7*	0.318	∗∗∗	-0.011	0.807	0.181	∗∗∗

Natural killer cell	*KIR2DL1*	-0.089	0.057	-0.158	∗∗∗	-0.072	0.123
	*KIR2DL3*	0.041	0.383	-0.130	∗∗	-0.027	0.562
	*KIR2DL4*	-0.025	0.586	-0.091	0.051	0.017	0.720
	*KIR3DL1*	0.023	0.621	-0.068	0.148	-0.009	0.853
	*KIR3DL2*	0.120	∗∗	-0.080	0.088	0.061	0.190
	*KIR3DL3*	-0.048	0.307	-0.091	0.051	0.010	0.825
	*KIR2DS4*	0.024	0.607	-0.114	∗	-0.048	0.305

Dendritic cell	*HLA-DPB1*	0.247	∗∗∗	-0.072	0.126	0.004	0.934
	*HLA-DQB1*	0.112	∗	-0.113	∗	-0.042	0.365
	*HLA-DRA*	0.118	∗	-0.158	∗∗∗	-0.054	0.245
	*HLA-DPA1*	0.201	∗∗∗	-0.129	∗∗	0.021	0.661
	*BDCA-1 (CD1C)*	0.308	∗∗∗	-0.102	∗	0.146	∗∗
	*BDCA-4 (NRP1)*	0.427	∗∗∗	-0.002	0.970	0.218	∗∗∗
	*CD11c (ITGAX)*	0.309	∗∗∗	0.020	0.668	0.146	∗∗

Th1	*T-bet (TBX21)*	0.197	∗∗∗	-0.098	∗	0.097	∗
	*STAT4*	0.171	∗∗∗	-0.226	∗∗∗	0.148	∗∗
	*STAT1*	0.184	∗∗∗	-0.037	0.435	0.107	∗
	*IFN-g (IFNG)*	-0.083	0.077	-0.133	∗∗	-0.015	0.746
	*TNF-a (TNF)*	0.079	0.092	-0.064	0.172	0.015	0.745

Th2	*GATA3*	0.336	∗∗∗	0.017	0.716	0.127	∗∗
	*STAT6*	0.361	∗∗	0.255	∗∗∗	0.337	∗∗∗
	*STAT5A*	0.375	∗∗∗	0.170	∗∗∗	0.232	∗∗∗
	*IL13*	0.034	0.473	0.019	0.685	-0.042	0.375

Tfh	*BCL6*	0.495	∗∗∗	0.172	∗∗∗	0.283	∗∗∗
	*IL21*	0.099	∗	-0.096	∗	0.042	0.365

Th17	*STAT3*	0.359	∗∗∗	0.045	0.336	0.396	∗∗∗
	*IL17A*	-0.161	∗∗∗	-0.134	∗∗	0.028	0.546

Treg	*FOXP3*	0.348	∗∗∗	0.020	0.664	0.145	∗∗
	*CCR8*	0.346	∗∗∗	-0.039	0.401	0.209	∗∗∗
	*STAT5B*	0.546	∗∗∗	0.277	∗∗∗	0.438	∗∗∗
	*TGFb (TGFB1)*	0.358	∗∗∗	0.045	0.340	0.088	0.060

T cell exhaustion	*PD-1 (PDCD1)*	0.173	∗∗∗	-0.045	0.337	0.053	0.254
	*CTLA4*	0.170	∗∗∗	-0.053	0.254	0.091	0.051
	*LAG3*	0.108	∗	-0.046	0.327	0.049	0.291
	*TIM-3 (HAVCR2)*	0.220	∗∗∗	-0.151	∗∗	0.052	0.266
	*GZMB*	0.019	0.684	-0.163	∗∗∗	-0.013	0.780

Note: ^∗^*P* < 0.05,  ^∗∗^*P* < 0.01, and^∗∗∗^*P* < 0.001.

## Data Availability

The data used to support the findings of this study are available from the corresponding author upon request.

## Supplementary Materials

Supplementary Materials Supplementary Figure 1: the PPI network and correlation analysis of core circadian clock genes in COAD.
