# Supplementary figures and images for: Circadian Clock Genes Are Correlated with Prognosis and Immune Cell Infiltration in Colon Adenocarcinoma
Source: Comput Math Methods Med. 2022 Jan 25;2022:1709918. doi: 10.1155/2022/1709918 (PMC8807038; doi:10.1155/2022/1709918)

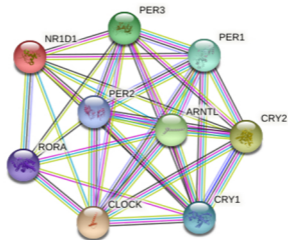

(a)

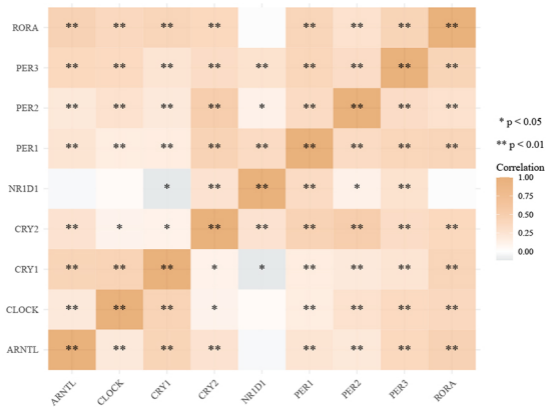

(b)

Supplement: Supplementary Materials — Supplementary Figure 1: the PPI network and correlation analysis of core circadian clock genes in COAD. [file 1709918.f1.pdf]
